# The Relationship between Paternal Alexithymia and Children’s Internalizing and Externalizing Behavioral Problems during Early Childhood

**DOI:** 10.3390/children10091498

**Published:** 2023-09-01

**Authors:** Donatella Scarzello

**Affiliations:** Department of Philosophy and Education Sciences, University of Turin, 10124 Turin, Italy; donatella.scarzello@unito.it

**Keywords:** alexithymia, fathers, internalizing and externalizing problems, parenting

## Abstract

The literature has long recognized that parental emotional competence, that is, the ability to express, understand, and regulate emotions, plays a key role in children’s development from early childhood. Nevertheless, the effect of parental alexithymia, which can be understood as a deficit in emotional competence, has not been thoroughly studied. In particular, the association between paternal alexithymia and behavioral problems in young children is still a neglected area of research. This study aims to investigate the association between paternal alexithymia and children’s internalizing and externalizing problems during the first three years of life, including whether overreactive parenting practices mediate the effect of alexithymia on children’s behavioral problems. A sample of 203 fathers of children aged 18–36 months were administered the TAS-20, the Overreactivity subscale of the Parenting Scale, and the Child Behavior Checklist (CBCL)/1½-5. The data indicate that paternal alexithymia is a predictor of children’s internalizing and externalizing behavioral problems and that paternal overreactivity mediates the effect of alexithymia. These results highlight the importance of preventing parental alexithymia and involving fathers in parenting support programs aimed at ensuring children’s mental health and adjustment.

## 1. Introduction

Children’s behavioral problems in the early childhood period impact future development and are associated with an increased risk of psychopathology [1,2,3] in later years. Assessing the factors behind children’s behavioral disorders is therefore particularly important from a prevention perspective. There are two main categories of behavioral problems: internalizing problems, such as anxiety, depression, social withdrawal, and somatic complaints, which are self-directed behaviors that cause intrapersonal distress, and externalizing problems, such as aggression and oppositionality, which are others-directed behaviors that cause conflict with the social environment. Both types stem from issues with emotional regulation skills [4,5,6], which are a crucial component of mental health and social adjustment [7,8,9].

### 1.1. Behavioral Problems, Children’s Emotional Regulation, and Parental Emotional Competence

The development of emotional regulation skills in children, critical for reducing the risk that behavioral problems will develop, depends on the interaction between personal characteristics (such as nervous system maturation and temperament) and experiences in the relational context [10,11]. In terms of the relational context, it should be kept in mind that children learn to express, understand, and regulate emotions primarily by observing and being supported by the parental model [12,13]. To be effective emotional socializers through the “emotion coaching” theorized by Gottman [14], parents must not only support children in emotional understanding and regulation, but first and foremost be themselves emotionally competent by being able to recognize, express, and regulate their own emotions [15].

Numerous studies confirm that issues of parental emotional regulation are associated with children’s emotional regulation issues [16,17], which in turn impacts their psychosocial adjustment and mental health.

Alexithymia is a deficit in emotional competence that has the potential to undermine parents’ emotional regulation processes and, therefore, their ability to be effective emotional socializers.

### 1.2. Alexithymia and Its Effects

Alexithymia is a deficiency characterized by difficulty identifying and communicating emotions, and a thinking style that is externally oriented and insufficiently introspective; it involves limited imaginative processes and difficulty engaging in cognitive processes of emotional regulation [18,19,20,21]. Although alexithymia was initially observed in individuals with psychosomatic disorders [22], it does not only affect those with psychiatric disorders; rather, it is also relatively common in the general population, appearing with a prevalence of about 10% [23,24,25].

Alexithymia is a considerable risk factor in the development of psychopathological conditions as it is associated with both internalizing and externalizing symptoms [26,27,28,29,30]. Recent studies on the impact of the pandemic have also found that individuals with alexithymic tendencies are more vulnerable to stressful events in that alexithymia plays a significant role in predicting the level of emotional symptoms and modulating the stress response [31,32,33].

The latest studies on alexithymia have adopted an interpersonal perspective, aiming to identify the effect of alexithymia on affective and social relationships. Alexithymia has been shown to negatively affect one’s ability to understand and relate to others; it is associated with poor social and affective skills, a low level of social support [34,35], and problems in couple relationships [36,37,38].

### 1.3. Parental Alexithymia and Parenting

Current research investigating the association between parental alexithymia and parenting quality appears promising. Alexithymic parents may have difficulty recognizing their children’s emotional signals and responding to them appropriately and proportionally, due to alexithymic traits that cause them problems in identifying their own and others’ emotional states. The literature on parental emotional socialization processes has shown that empathic, validating, and comforting responses to children’s negative emotions promote children’s exploration of emotions, resulting in greater emotional awareness and regulatory skills. Conversely, parental behaviors aimed at ending negative emotional experience (e.g., distracting children, distancing themselves from the child, belittling children’s emotions, or punishing them) may impair children’s opportunities to identify and label emotions and to learn emotion regulation skills. In fact, these parental behaviors have been found to be associated with an increased risk of internalizing and externalizing behaviors in children across different age groups [39,40].

Parental alexithymia can adversely affect the parent’s ability to ‘be with’ the child’s emotions. As described by attachment theory, such ‘being with’ involves the parents being attuned to the child’s emotions, and it is essential to effectively act as an emotional coach for children and foster their emotional competence. Indeed, some studies show that alexithymia can have a negative effect on parenting by decreasing sensitivity, responsiveness, mentalization, and parental empathy, and by promoting the establishment of insecure attachment patterns [41,42,43].

Alexithymia is also associated with higher levels of parenting stress [44] and dysfunctional discipline practices, such as those characterized by excessive emotional reactivity [45]. Alexithymic individuals have trouble controlling emotional arousal, which impedes conflict management by increasing the likelihood of aggressive acts [46]; it can lead parents to manage the stress associated with the parenting role poorly and react to their children angrily and impulsively.

With a few exceptions [29,41,44,45], existing studies on the association between parental alexithymia and parenting focus predominately on the mother figure, and this association requires further investigation, as it is still an under-researched area.

### 1.4. Parental Alexithymia, Children’s Emotional Dysregulation, and Internalizing and Externalizing Problems

As mentioned above, parental emotional competence has a significant impact on children’s emotional competence. Emotional regulation is an aspect of emotional competence that has been widely studied, and various authors suggest that it is transmitted from generation to generation [47]. Some authors hold that alexithymia can also be transmitted intergenerationally [43], which is explained in light of the difficulties alexithymic parents face in socializing emotions [48]. In some research investigating samples of different ages, parental alexithymia correlated with that of children [49,50,51]. However, not all results confirm this hypothesis [29,52], or studies confirm it only partially for some age groups [53].

Some studies have focused on whether there is an association between parental alexithymia and internalizing and externalizing symptoms in children, as the emotional regulation component is central to these problems. However, the results are inconclusive [54]. Parental alexithymia plays a role in predicting children’s maladjustment, but it is unclear how parental alexithymia specifically affects internalizing and externalizing symptoms. A number of studies have shown that internalizing emotional symptoms in children are associated with parental alexithymia [55,56]. While some studies have found that externalizing symptoms are related to parental alexithymia [29], others have not [55].

### 1.5. The Current Study

As argued by Arhneberg and colleagues [41], research on parental alexithymia almost always considers only mothers. This study thus aims to help fill the gap by focusing on fathers. The literature has long highlighted the specific role fathers play in child development and psychosocial outcomes [57,58,59,60,61,62]. Alexithymia in fathers may be an interesting focus of investigation in that research indicates that men have more difficulty than women identifying and communicating feelings [45,63]. This is likely due to stereotypical cultural expectations that cause men to hide and suppress their emotions in order to appear strong. Our research focuses on children in the first three years of life, because the available studies mainly focus on the effect of parental alexithymia on older children [29,54,55,56]. The effect of parental alexithymia may be particularly evident during early childhood and is therefore a significant risk factor to monitor. In fact, children in this age group in particular need the support of significant adults to regulate their emotions and behaviors [64,65].

The study has the following specific objectives:

1. To analyze the role of paternal alexithymia in children’s internalizing and externalizing behavioral problems (as evaluated by fathers) during early childhood. Specifically, whether paternal alexithymia has a different and distinct effect on internalizing and externalizing problems. Previous studies are contradictory, especially regarding the effect on externalizing problems.
2. To identify which of the three main features of alexithymia in fathers (difficulty identifying feelings, difficulty communicating feelings, and externally oriented thinking) are most involved in influencing the internalizing and externalizing problems of children as reported by fathers. The literature suffers from a lack of data on this issue.
3. To investigate whether dysfunctional paternal parenting practices, characterized by overreactivity, mediate the relationship between alexithymia and children’s behavioral problems. Fathers with higher levels of alexithymia may enact behaviors characterized by anger and aggression more frequently when faced with situations in which children disobey rules and exhibit undesirable behaviors, as indicated in previous studies [45]. In turn, reacting in an uncontrolled manner in daily educational interactions with their children limits the children’s opportunity to observe effective models of emotion and behavior regulation. It is thus hypothesized that the effect of paternal alexithymia on children’s behavioral problems is not only direct, but also indirect and that, in the latter case, it is mediated by overreactive parenting practices.

## 2. Materials and Methods

### 2.1. Participants

The research subjects were 203 fathers, mostly of Italian origin (98.5%), parents of children aged 18–36 months (mean age = 24.69; SD = 7.585), 51.7% male and 48.3% female. The fathers had a mean age of 37.65 years (SD = 5.179; range: 24–55). Socioeconomic status (SES), as measured with Hollingshead’s index (1975), which takes into account both schooling and current occupation, was upper-middle class (M = 42.24; SD = 16.61; range 11–66). Almost all fathers worked full-time (94.3%); 68% were married, 27% were cohabiting, and 5% were separated/divorced. Of the total, 67% had only one child; where the fathers had multiple children, questionnaires were completed referring to the child aged 18–36 months (children under 18 months were excluded from the sample because of the instruments chosen, which will be presented in the following section).

### 2.2. Procedure

The study was conducted in compliance with Italian data protection regulations and in accordance with the ethical standards of the American Psychological Association (APA), with approval received from the University Ethics Committee. Ten nursery schools in a large northern Italian city (Turin) were contacted for the research and chosen so as to ensure a heterogeneous social and economic pool. The link to the online Google Form was given by the nursery schools, through their managers and educators, to the fathers of children using their services. The introduction on the form explained that participation in the study was voluntary and data would be collected anonymously. The criterion for inclusion in the study was that respondents be fathers with at least one child between the ages of 18 and 36 months.

### 2.3. Measures

The online questionnaire consisted of 4 sections: socio-demographic information; the Toronto Alexithymia Scale (TAS-20); the Overreactivity subscale of the Parenting Scale; and the Child Behavior Checklist (CBCL)/1½-5.

#### 2.3.1. Socio-Demographic Information

An ad hoc questionnaire was formulated to collect information such as age, nationality, marital status, education level, occupation, family structure, and gender and age of the child.

#### 2.3.2. Paternal Alexithymia

The fathers filled out the Toronto Alexithymia Scale [66,67], which consists of 20 statements for which the subject indicates his degree of agreement via a 5-point Likert scale (from 1 = ‘I do not agree at all’ to 5 = ‘I completely agree’). The instrument is divided into three subscales: difficulty identifying feelings (DIF, 7 items); difficulty describing feelings (DDF, 5 items); and externally oriented thinking (EOT, 8 items).

The higher the scores, the greater the degree of alexithymia. The clinical cutoff is 61, a threshold that identifies alexithymic subjects. Total scores below 50 refer to the absence of alexithymic traits, while values between 50 and 60 indicate an intermediate score [21]. The psychometric properties of the scale in its Italian adaptation have also been confirmed in numerous studies [67,68,69] with Cronbach’s alpha coefficients ranging from 0.51 (for the EOT scale) to 0.79.

#### 2.3.3. Paternal Overreactivity

The Overreactivity subscale of the Parenting Scale [70] was used to identify the presence of dysfunctional parenting practices characterized by excessive emotional reactivity. The Parenting Scale is a self-reporting measure that investigates the use of dysfunctional parenting practices in situations requiring discipline. The questions consider potentially confrontational situations in which the child displays undesirable behaviors that test parental abilities to express, understand, and manage emotions in relational contexts.

The Overreactivity subscale measures parental anger and irritability behaviors associated with an authoritarian behavior style. It consists of 10 items on a 7-point Likert scale, where 7 indicates a high likelihood of committing discipline errors and 1 suggests the likelihood of using an alternative, effective discipline strategy. A higher score therefore indicates a more dysfunctional disciplinary style. The reliability of the instrument appears to be high (Cronbach’s alpha = 0.80).

#### 2.3.4. Children’s Behavioral and Emotional Problems

To explore the presence of internalizing and externalizing behavioral problems, this research used the Child Behavior Checklist (CBCL)/1½-5 [71], which includes 100 statements about specific problems. Fathers rated the prevalence of each problem on a three-point Likert scale (0 = not true; 1 = sometimes true; 2 = very/often true). For this study, the Internalizing Problems (36 items) and Externalizing Problems (24 items) subscales were used.

### 2.4. Data Analysis

Statistical analyses were performed using the Statistical Package for Social Science version SPSS 28 for Windows. First, descriptive analyses of the variables considered were carried out, and, in the case of TAS-20, the number of subjects at risk was identified according to normative criteria. Variance analysis was performed to explore if there was a gender difference in the level of children’s behavioral problems as assessed by fathers. Next, Pearson’s correlation analysis was calculated to measure any association between sociodemographic variables and the variables under consideration: in the case of fathers, the association between age, SES, and paternal alexithymia and overreactivity; in the case of children, the association between age and degree of behavioral problems. In the case of overreactive parenting, a variance analysis was also carried out to reveal possible differences between fathers with one child and those with more than one child.

Associations between children’s behavioral problems and paternal alexithymia were then explored through Pearson’s correlation analysis and variance analysis (considering two groups of fathers, in this case: those at risk and those not at risk for alexithymia, according to the TAS-20 cutoff). Multiple linear regressions were then performed to assess the relationship between various characteristics of paternal alexithymia (difficulty identifying feelings, difficulty communicating feelings, externally oriented thinking) and children’s behavioral problems.

Finally, the mediation hypothesis was tested using the Sobel test, including alexithymia as a predictor, children’s behavioral problems as a dependent variable, and paternal overreactivity as a mediating variable.

## 3. Results

### 3.1. Paternal Alexithymia

Table 1 shows the results of the descriptive analyses performed on the TAS-20 results.

The average levels of fathers’ alexithymia are in line with those reported in other Italian studies [29,67]. The scores were also analyzed according to the scale’s normative data: 73.2% of fathers have no alexithymic traits (score below 50), 21.8% are in the intermediate level (scores between 50 and 60), and 5% of fathers are at risk (score above 60).

The alexithymia scores do not appear to be associated with either socioeconomic status (SES) or age, except for a weak positive correlation between age and externally oriented thinking (r = 0.198; *p* < 0.01).

### 3.2. Paternal Overreactivity

Table 2 shows the results of the descriptive analyses performed on the results of the Overreactivity subscale of the Parenting Scale.

Correlation analysis shows a positive association between the frequency of overreactivity and fathers’ age (r = 0.307; *p* < 0.001) as well as children’s age (r = 0.254; *p* < 0.001), while no differences were found with respect to paternal SES and children’s gender.

The variance analysis indicates a significant difference in overreactivity between fathers with one child and those with multiple children (F = 17.873; *p* < 0.001); fathers with multiple children engage in overreactive parenting more frequently (mean = 3.2; SD = 0.56) than those with one child (mean = 2.8; SD = 0.6).

### 3.3. Children’s Problematic Behaviors

Table 3 shows the results of the descriptive analysis performed on the CBCL filled out by fathers.

The variance analysis shows no differences in terms of children’s gender.

Some positive correlations related to age were found: older children, in fathers’ perceptions, have more issues in both scales, internalizing (r = 0.299; *p* < 0.001) and externalizing (r = 0.209; *p* < 0.005) problems.

### 3.4. Relationship between Paternal Alexithymia and Children’s Behavioral Problems

Table 4 shows the correlations between paternal alexithymia and internalizing and externalizing problems in children.

The total alexithymia score and subscales from the TAS-20 correlated significantly with the Internalizing and Externalizing Problems subscales, although correlations with the DCF subscale (difficulty communicating feelings) were weak.

To investigate these aspects in more depth, a variance analysis was carried out by considering the clinical cutoff of total TAS-20 scores: fathers who fall into the “at-risk” category for alexithymia (scores above 60) have children with significantly higher scores in the subscales for both internalizing (F = 119.12; *p* < 0.001) and externalizing problems (F = 15.121; *p* < 0.001).

### 3.5. Relationship between Characteristics of Paternal Alexithymia and Children’s Behavioral Problems

To clarify which characteristics of paternal alexithymia have a stronger effect on the children’s various behavioral problems, a series of multiple regression analyses with the stepwise method were performed with the scores from the CBCL Internalizing and Externalizing Problems subscales as dependent variables and the scores of the TAS-20 subscales—DIF (difficulty identifying feelings), DCF (difficulty communicating feelings), and EOT (externally oriented thinking)—as predictor variables.

The data show that a significant predictor of both subscales is difficulty identifying feelings (internalizing problems: ß = 0.331; t = 3.823; *p* < 0.001; externalizing problems: ß = 0.354; t = 4.768; *p* < 0.001); for internalizing problems, externally oriented thinking is also a predictor (ß = 0.182; t = 2.104; *p* < 0.05).

### 3.6. Analysis of Mediation

We then proceeded to investigate the relationship between the variables under consideration in more detail, hypothesizing that alexithymia is a predictor of both children’s behavioral problems and paternal overreactivity, and that alexithymia also has an indirect effect on behavioral problems, mediated by overreactivity. We thus began by examining the unique contribution of alexithymia in explaining variance in internalizing and externalizing behavioral problem scores. Two regression analyses with the stepwise method were carried out with the TAS-20 total score as predictor variable and the scores of internalizing and externalizing behaviors as dependent variables. The data show that alexithymia predicts both internalizing (ß = 0.406; t = 5.474; *p* < 0.001) and externalizing (ß = 0.340; t = 4.565; *p* < 0.001) behavioral problems.

The study subsequently tested the relationship between alexithymia and overreactivity through a linear regression analysis with the TAS-20 total score as the independent variable and the overreactivity score as the dependent variable. The findings confirmed that alexithymia predicts overreactivity (ß = 0.45; t = 6.322; *p* < 0.001).

Regression analysis confirmed that overreactivity meets the requirements for mediation since, in addition to being predicted by alexithymia as outlined above, it in turn predicts children’s behavioral problems, both internalizing (ß = 0.432; t = 5.888; *p* < 0.001) and externalizing (ß = 0.472; t = 6.737; *p* < 0.001).

We therefore conducted two mediation analyses by including the TAS-20 total score as the independent variable and overreactivity as the mediating variable and setting internalizing problems and externalizing problems as dependent variables. The Sobel test confirmed the hypothesis that overreactivity mediates the relationship between alexithymia and behavioral problems, both internalizing (Sobel test = 3.76; *p* < 0.001) and externalizing (Sobel test = 4.3; *p* < 0.001).

In the case of externalizing problems, paternal alexithymia seems to have only an indirect effect, as the inclusion of overreactivity in the model cancels out the predictive value of alexithymia (ß = 0.132; t = 1.694; *p* = n.s. 0.092); in this case, therefore, full mediation is demonstrated. For internalizing problems, even with the inclusion of overreactivity, alexithymia continues to be a significant predictor of the degree of internalizing problems (internalizing problems: ß = 0.234.; t = 2.942; *p* < 0.005); the mediation effect is therefore partial in this case.

## 4. Discussion

There are limited data available on paternal alexithymia and its relationship to children’s behavioral problems, although it is generally known that parental emotional competence is crucial in supporting children’s development, especially during the age group of early childhood analyzed here.

The first objective of this research was to investigate the role of paternal alexithymia in influencing children’s internalizing and externalizing behavioral problems during early childhood (as perceived by fathers).

Previous studies are in agreement that parental alexithymia does have an identifiable effect on children’s internalizing problems [55,56], while the findings regarding associations between parental alexithymia and children’s externalizing problems are inconsistent [29,55].

Our data indicate that paternal alexithymia is associated with both categories of problems. This pervasive effect of alexithymia may be due to the specific age group considered in our study, namely very young children. Indeed, children are particularly in need of parental support for emotional and behavioral regulation in the early years of life, and the lack of such support could result in both internalizing and externalizing symptoms.

The findings are in line with research conducted in recent decades highlighting the important role played by fathers in influencing children’s development [59], and with the literature stressing that parents, in their capacity as emotional socializers, influence children’s emotional and behavioral regulation skills. Parents who exhibit a limited ability to understand their own and others’ emotions and consequently poor empathic responses—as is the case with alexithymic individuals—do not foster a sufficient development of regulation skills in their children [64].

The second objective was to pinpoint which dimensions of paternal alexithymia are specifically involved in influencing children’s internalizing and externalizing problems. Analysis of the data showed that the most salient dimension for both internalizing and externalizing problems was difficulty identifying feelings. Previous studies using the TAS-20 have also found that the dimension most closely associated with problems in relationships with children is difficulty identifying feelings, as this aspect implies a reduced empathic capacity, which in turn results in decreased sensitivity and responsiveness [72] and the enactment of dysfunctional parenting practices [45].

Our data also indicate that, in addition to difficulty identifying feelings, externally oriented thinking in fathers also plays an important role in predicting children’s internalizing behaviors. In line with these findings, previous studies have shown that this dimension is significant in the case of fathers—but not mothers—in influencing affective and educational relationships with their children [41,45]. A cognitive style focused on concrete aspects of external reality instead of inner processes is more commonly found in men than in women [41,45], and this might account for the importance of this dimension in fathers. In addition, in the context of emotional socialization processes, such a cognitive style could translate into behaviors intended to distract children from their emotional experience and quickly bring it to an end, for example, by redirecting children’s attention and engaging them in hands-on activities without giving them time to reflect on what is going on inside them. Parental responses of this kind are defined by the literature as outcome-oriented rather than process-oriented [73] and, as some studies indicate, they result in children enacting self-oriented regulatory strategies such as emotion suppression [74]. The latter is indeed the regulatory strategy most typically found in the case of internalizing behaviors.

The third objective was to analyze whether paternal overreactive parenting mediates the relationship between alexithymia and children’s behavioral problems. The results confirm this mediating effect: alexithymia affects overreactivity, which in turn influences children’s behavioral problems. Emotional awareness and processing issues hamper fathers’ regulation of negative emotions during challenging educational situations, such as those in which the child transgresses rules, resulting in the enactment of overreactive educational practices. In turn, paternal overreactivity increases the risk of behavioral problems in children because the way in which the adult expresses and regulates negative emotions presents an opportunity for the child to learn through model observation [10,11]. Indeed, children are likely to emulate parental regulation strategies as well as behaviors characterized by dysregulation [75].

In the case of externalizing problems, overreactivity fully mediates the relationship between alexithymia and children’s symptoms. In the case of internalizing problems, the mediation is only partial: the direct effect of alexithymia on internalizing symptoms remains. Although this result will need to be further investigated in subsequent studies, it may at least partially explain the result found in some previous research that parental alexithymia predicts children’s internalizing symptoms but not their externalizing ones [55].

This research also offers some interesting findings related to paternal overreactivity. Indeed, the data show that fathers tend to be more reactive in cases where they have more than one child. Managing the needs and demands of multiple children at the same time is more complex and tends to increase levels of parental stress, as also found in other studies [76]. In addition, there is a correlation between paternal overreactivity and children’s age. The analysis of the CBCL data found that, according to fathers’ assessments, it is actually the older children who display higher degrees of both internalizing and externalizing problems, and this may be why their behaviors pose a greater challenge to fathers, who are more frequently inclined to react with impulsivity and anger. In addition, it may be more difficult to remain calm in the face of older children’s transgressions and oppositional behaviors, because parents expect them to be more able to adhere to acceptable behaviors than younger children. Breaking with this expectation thus results in greater levels of disappointment and anger. This hypothesis is supported by a study by Cole and colleagues, who, in longitudinal research, examined the impact of children’s developmental level for children aged 18 to 48 months on maternal emotions: it was found that children’s oppositional and disruptive behaviors do not elicit negative maternal emotions when the children are 12, 24, and 36 months old but do when the children are 48 months old, because parents expect children to be able to self-regulate as they grow older [77].

## 5. Limitations and Future Research

This research entails some limitations: as the study is cross-sectional and not longitudinal, it is not possible to ascertain whether the association between paternal alexithymia and behavioral problems is a cause-and-effect one.

In addition, given the use of only self-reporting on the part of fathers, it is not possible to say with certainty that alexithymia increases the frequency of children’s behavioral problems: it could also be hypothesized that fathers with higher levels of alexithymia perceive higher levels of behavioral problems in their children, due to a difficulty in recognizing the children’s emotions and correctly interpreting maladaptive symptoms.

In future studies, therefore, it would be worthwhile to adopt a longitudinal research design and either pair measures such as the CBCL filled out by fathers with observational methods for scoring children’s behavior or use a multi-informant method, with the CBCL (or similar instruments) filled out for each child not only by the father but also by the mother or other significant adults such as caregivers and teachers.

Because the children’s mothers were not considered in this research, we cannot know how maternal characteristics influence the relationship between paternal alexithymia and children’s adjustment; this aspect should be explored in future studies.

Finally, in subsequent research, it would also be valuable to consider child characteristics, such as the child’s emotional regulation ability and temperament, as these strongly affect emotional and behavioral adjustment and could mediate the impact of parental characteristics on developmental outcomes. Since psychopathological risk can be viewed as the result of a dynamic interplay between genetic and environmental risk factors [78] and much recent research adopts complex models of psychopathology that incorporate a focus on gene–environment and epigenetics interactions [79,80,81], the child’s genetic characteristics could also be analyzed to better understand the effects of paternal characteristics on children’s behavioral problems.

## 6. Conclusions

The results clearly indicate that alexithymia, and not only the maternal alexithymia that has been most commonly studied in the literature but also the paternal alexithymia considered in this study, can constitute an obstacle to children’s adjustment during early childhood. It is therefore crucial to design initiatives aimed at preventing alexithymia in fathers by focusing on fostering their ability to recognize, express, and regulate emotions.

Parenting support strategies should promote paternal skills in terms of regulating negative emotions even in the face of challenging educational situations in order to avoid resorting to overreactive parenting practices since, as pointed out in the literature and confirmed by our data, such practices contribute to the development of behavioral problems in children.

Careful monitoring of the risk of parental alexithymia and early interventions involving both mothers and fathers could thus counteract the risk of maladaptive developmental outcomes in children and perhaps even counteract what some authors have termed the intergenerational transmission of alexithymia [43].

## Figures and Tables

**Table 1:** Tas-20.

TAS 20	M	SD	Range
TOTAL SCALE	42.23	10.97	20–63
DIF Difficulty Identifying Feelings	11.25	4.06	7–20
DDF Difficulty Describing Feelings	12.23	4.29	5–21
EOT Externally Oriented Thinking	18.81	5.45	8–30

**Table 2:** Parenting Scale.

Parenting Scale	M	SD	Range
Overreactivity	29.6	6.17	18–42

**Table 3:** CBCL.

CBCL	M	SD	Range
Internalizing	5.80	5.43	0–23
Externalizing	8.53	6.23	0–27

**Table 4:** Correlations.

	CBCL Internalizing Scale	CBCL Externalizing Scale
TAS 20-TOTAL	0.406 **	0.340 **
TAS 20-DIF	0.462 **	0.387 **
TAS 20-DCF	0.181 *	0.195 *
TAS 20-EOT	0.370 **	0.284 **

* *p* < 0.05 (two-tailed), ** *p* < 0.001 (two-tailed).

## Data Availability

The datasets generated and/or analyzed in the current study are available from the corresponding author upon reasonable request.
